# Engineering Efferocytosis for Bone Regeneration

**DOI:** 10.1002/mabi.202500094

**Published:** 2025-07-04

**Authors:** Jacob Miszuk, Linna Zhong, Hongli Sun

**Affiliations:** ^1^ Department of Restorative Sciences & Biomaterials Boston University Henry M. Goldman School of Dental Medicine Boston Massachusetts USA; ^2^ Iowa Institute for Oral Health Research University of Iowa College of Dentistry Iowa City Iowa USA; ^3^ Department of Oral and Maxillofacial Surgery University of Iowa College of Dentistry Iowa City Iowa USA

**Keywords:** biomaterials, bone regeneration, drug delivery, efferocytosis, tissue engineering

## Abstract

Bone is an incredibly robust tissue thanks to its high blood supply, rapid cell turnover, and continuous remodeling. A significant body of research investigates strategies to improve osteogenesis, angiogenesis, and immunomodulation for bone regeneration, facilitated by numerous various therapeutic approaches (e.g. pharmacologics, biomaterials, stem cell therapy, and more). However, a critically understudied but recently emerging area of research lies in the inflammatory cascade and the cleanup of apoptotic cells during repair, aging, and disease. Termed “efferocytosis,” this natural and efficient cleaning up of cells at the end of their lifespan is a crucial step in resolving injury, controlling disease, maintaining homeostasis, and tissue repair. Currently, the primary mechanism(s) driving efferocytosis in most tissue but especially bone, is unknown. Despite this knowledge gap, mounting evidence suggests that impaired efferocytosis plays a significant role in many chronic illnesses and impairs tissue regeneration. Biomaterials‐based interventions are well‐positioned to interrogate mechanisms of efferocytosis due to their ability to provide local support and guide cellular activity not only in combination with but also without additional pharmaceutical aid. This review will highlight the current understanding of efferocytosis in bone and discuss cutting‐edge biomaterials‐based strategies to engineer efferocytosis for improved outcomes in bone regeneration.

## Graphical abstract

The repair of bone tissue in acute and chronic injuries is a significant long‐standing clinical challenge. Biomaterials‐based interventions are well‐positioned to interrogate mechanisms of efferocytosis due to their ability to provide support and guide cellular activity. This review will highlight the current understanding of efferocytosis in bone and discuss cutting‐edge biomaterials‐based strategies to engineer efferocytosis for improved outcomes in bone regeneration.

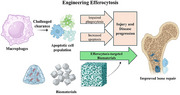

## 1 Introduction

The repair of bone tissue in acute and chronic injuries is a significant long‐standing clinical challenge. Regenerative medicine‐assisted strategies engineer a niche of scaffolds, bioactive molecules, and stem cells to recover and replace damaged or lost tissue [1, 2]. Recently, great attention has been paid to the role of the inflammatory response in this regeneration cascade, as it is found to play a key role in guiding the healing process [3, 4]. Under normal homeostasis conditions, apoptotic cells are continually cleared and removed from tissue so that normal body function can continue – a process called “efferocytosis” [5, 6]. These apoptotic cells are cleared/removed by various phagocytes, such as “professional” cells like macrophages and osteoclasts in bone, or “non‐professional” cells like liver epithelial cells or fibroblast/kidney cells in other tissue [7, 8, 9]. During disease, trauma, and aging, this clearance of apoptotic cell populations can be impaired, leading to dysfunction or furthering of chronic disease and cancer. Recent evidence in literature suggests that facilitation of this mechanism can be a significant driver in improved outcomes in the regeneration of bone and other tissues [10, 11].

While many connections have been posited about the role of efferocytosis in bone regeneration, the main driving mechanisms are still unclear. Dysregulation of efferocytosis can derive from intrinsic and environmental factors, and affect one or more of phagocytes, apoptotic cell populations, or other associated biological machinery. Therapeutic interventions for efferocytosis are a downstream goal, but many involved mechanisms must be uncovered and understood before these opportunities can be realized. This review will cover the recent findings and advances in engineering efferocytosis to improve outcomes in bone regeneration, and what questions lie ahead on the path toward uncovering more of the inner workings of efferocytosis.

## 2 Efferocytosis: A Background

Billions of cells in the body undergo programmed death via apoptosis every day, where a coordinated biological process removes unneeded or damaged cells for various reasons. In most cases, cells undergoing apoptosis go through a well‐characterized process beginning with outer membrane permeabilization that leads to eventual cellular disintegration [12]. Buildup and accumulation of apoptotic debris often leads to progression and complications of chronic disease, making it essential that they are promptly dealt with by the biological machinery of the body. During this process of dying, cells communicate with their surroundings in manners such as physical contact with neighboring cells [13] or release of soluble factors into surrounding tissue [14, 15, 16], which helps to guide an appropriate phagocyte to eat the soon‐to‐be dead cell. The leftover cellular components are then swiftly engulfed by those phagocytes, triggering changes that typically lead to pro‐resolving phenotypes to combat inflammation. This process is known as efferocytosis – an organized system of distinct phases encompassing cell signaling, phagocytic migration, and recognition, and eventual digestion. This process is tightly coupled and highly efficient, as observation of apoptotic cell populations in vivo is extraordinarily difficult [17].

Efferocytosis is typically carried out by the primary “professional” phagocytes ‐ macrophages [18, 19]. Found in nearly all tissue types, macrophages are a highly mobile and plastic cell population capable of adapting to the body's needs to induce or combat inflammation. The macrophage's role in efferocytosis has been highlighted in various pathologies [20, 21, 22, 23], indicating a significant contribution to inflammatory and disease resolution. However, in some tissue types, “non‐professional” cells can contribute to the efferocytosis step and help with the cleanup of apoptotic debris as well. Examples of these include mesenchymal stromal cells in bone marrow [24], epithelial cells in the intestine [25], and hair follicle stem cells [26]. The impact of non‐professional cells' contribution to overall efferocytosis in tissue is, to date, still largely unclear.

A dysregulate efferocytosis is implicated in numerous pathologies as it has been noted in atherosclerosis [27], diabetes [28], rheumatoid arthritis [29], and aging. It is imperative that we gain a stronger understanding of the mechanisms governing efferocytosis in specific tissues across the body so we may develop therapeutics tailored to combat disease and improve quality of life in aging.

## 3 Efferocytosis in Bone

Efferocytosis in bone is carried out by a variety of cells due to its highly heterogenous cell population and high cell turnover relative to most other tissues in the body [24, 30]. Remodeling is a constant dance of osteoblastic and osteoclastic activity that both deposits and removes bone, and as such, generates a significant number of apoptotic cells during these processes. It is suggested that a significant number of osteoblasts terminal fate through the apoptosis pathway rather than osteocyte or bone lining cells [31]. Studies on the overexpression of various apoptosis inhibitors have shown inconsistent results in promoting osteoblastic differentiation [32, 33]. This has led to unclear mechanisms of how efferocytosis is recognized in osteoblasts. Some recent evidence suggests that Sirtuin 6 (SIRT6) protein—a protein heavily involved in DNA repair—plays a role in macrophage recruitment for efferocytosis [34] and inflammation resolution [35], but more studies are needed to further elucidate this mechanism. Receptor Mer proto‐oncogene tyrosine kinase (MerTK) is one key player in the maintenance of bone homeostasis [36]. MerTK is shown to play a notable role in efferocytosis, as evidenced by aggravated arthritis via cartilage and bone erosion [37]. Calcium signaling plays a significant role in MerTK‐mediated efferocytosis, as intracellular calcium levels are critical to the internalization of cellular payloads [38]. This feature is intriguing as the bulk of the body's calcium is stored in bone, and MerTK‐mediated efferocytosis is also scrutinized in other tissues [39, 40].

While some signaling pathways are becoming identified as major players in efferocytosis, many questions are still unanswered with regard to phagocytic activity in bone due to its highly heterogenous cell traffic and turnover. Do osteoblasts wholly undergo apoptosis or have divergent cell fates? Are all apoptosis targets cleared by a singular phagocyte type? What is the extent of the contribution of mesenchymal stromal cells and other non‐professional cells to efferocytosis in bone, and how does it change with age and other various pathologies? Many of the current known targets are highlighted in Table 1. Homing in on these answers will provide more direct and clear avenues for therapeutic strategies that will help us improve therapeutic outcomes in advancing age and disease in the clinic.

**TABLE 1:** Known efferocytes in bone, their known targets, and potential targets for phagocytic uptake.

Phagocytes in bone	Known target(s)	Potential targets
Bone marrow macrophages	Osteoblast [41], Osteoclast [42], Neutrophils [43]	Erythroblasts [44], hematopoetic stem cells [45]
Osteal macrophages (Osteomacs)	Osteoblast [46, 47]	Unknown
Osteoclasts	Osteocytes [48], Neutrophils [49]	Chondrocytes [11]
Septoclasts	Chondrocytes [50]	Unknown
Mesenchmyal stromal cells	Neutrophils [24]	Unknown

### 3.1 Acute Trauma

Injury and trauma to bone bring consequences to both macrophage and apoptotic cell populations. Trauma causes an increase in apoptotic cell population near the site of the incident, due directly to the injury/trauma or result of disrupted blood supply [51, 52]. Macrophage recruitment to injury site is a crucial step in the resolution of injury, especially for the cleanup of dead/apoptotic cells [53]. Impediment of macrophage mobility during acute trauma or ablation of macrophages shows decreased tissue resolution and impaired bone union during healing [54]. While macrophage activity and apoptotic cell population are clearly altered during injury, the mechanisms driving the change in their behavior is still unclear—whether it significantly impacts efferocytosis or not.

### 3.2 Chronic Injury/Aging Environment

Regenerative capacity of bone tissue is reduced with age due to a number of factors, among them is a change in the activity levels of macrophages [55, 56]. Aging is also tightly associated with a buildup of senescent cell population, resulting in delayed healing and increased resorption of bone [57, 58]. Low‐density lipoprotein receptor‐related protein 1 (Lrp1) levels are significantly reduced in macrophages in aged mice and humans, leading to a reduced rate of fracture repair in old mice [59], where evidence strongly suggests Lrp1 is a critical mediator for macrophage‐mediated efferocytosis in various tissues [60, 61]. Aging is also tightly associated with the prevalence of osteoporosis, where literature examining efferocytosis during osteoporosis is nearly nonexistent. Some evidence suggests high glucose conditions of diabetic osteoporosis inhibit efferocytosis in a diabetic rat model [49], but very little details about efferocytosis in non‐co‐morbidities are known. Reduced estrogen levels are strongly linked to osteoporosis, especially in post‐menopause. Other evidence in microglial cells suggests that estrogen regulates phagocytic clearance of apoptotic cells in inflammatory conditions [62], but so far its role in bone efferocytosis is relatively unexplored. Additionally, while phagocytic activity sees shifts during aging, another contributing factor is the change in the apoptotic osteoblast population. Aged mice are found to display an increased expression of CD47, leading to evasion of immune surveillance and clearance by macrophages [34]. As the world's population continues to age and life expectancy rises, uncovering these age‐related changes of efferocytosis could become crucial in guiding therapeutic development for the future of medicine.

### 3.3 Periodontitis

A disordered resolution of inflammation is considered a key feature of periodontitis, where a chronic lack of clearance of apoptotic neutrophils eventually leads to breaking down and destruction of gingival tissue [63, 64]. Unresolved chronic inflammation in periodontitis leads to local bone loss, and is strongly linked to many other diseases such as atherosclerosis [65], diabetes mellitus [66], chronic kidney disease [67], and other various cardiovascular diseases [68], making it a key point of study in the inflammatory cascade. As it is currently understood, efferocytosis in periodontal tissue is normally carried out by macrophages. Pro‐healing M2 macrophages are generally more in number than pro‐inflammatory M1 macrophages in healthy tissue [69], but the reverse is the case in periodontitis [70, 71]. Developmental endothelial locus‐1 (DEL‐1) is an emerging player in periodontal inflammation, where it is shown that DEL‐1 protein concentration is upregulated in periodontal inflammatory clearance in humans [72]. Recombinant DEL‐1 administration resulted in a significant reduction in apoptotic neutrophils in mice, suggesting one of its roles is increasing efferocytosis. Li et al. demonstrate that the DEL‐1 and CD36 efferocytosis‐related molecules are modulated via SIRT6‐miR‐216/217 axis in a diabetic periodontitis model, where SIRT6 overexpression resulted in increased M2 macrophage polarization and downstream efferocytosis [35]. Further upstream factors are still pending investigation in the impact of efferocytosis in periodontitis, but are undoubtedly potentially promising targets for therapeutic interventions in future research.

## 4 Therapeutic Targets for Efferocytosis

**FIGURE 1:**
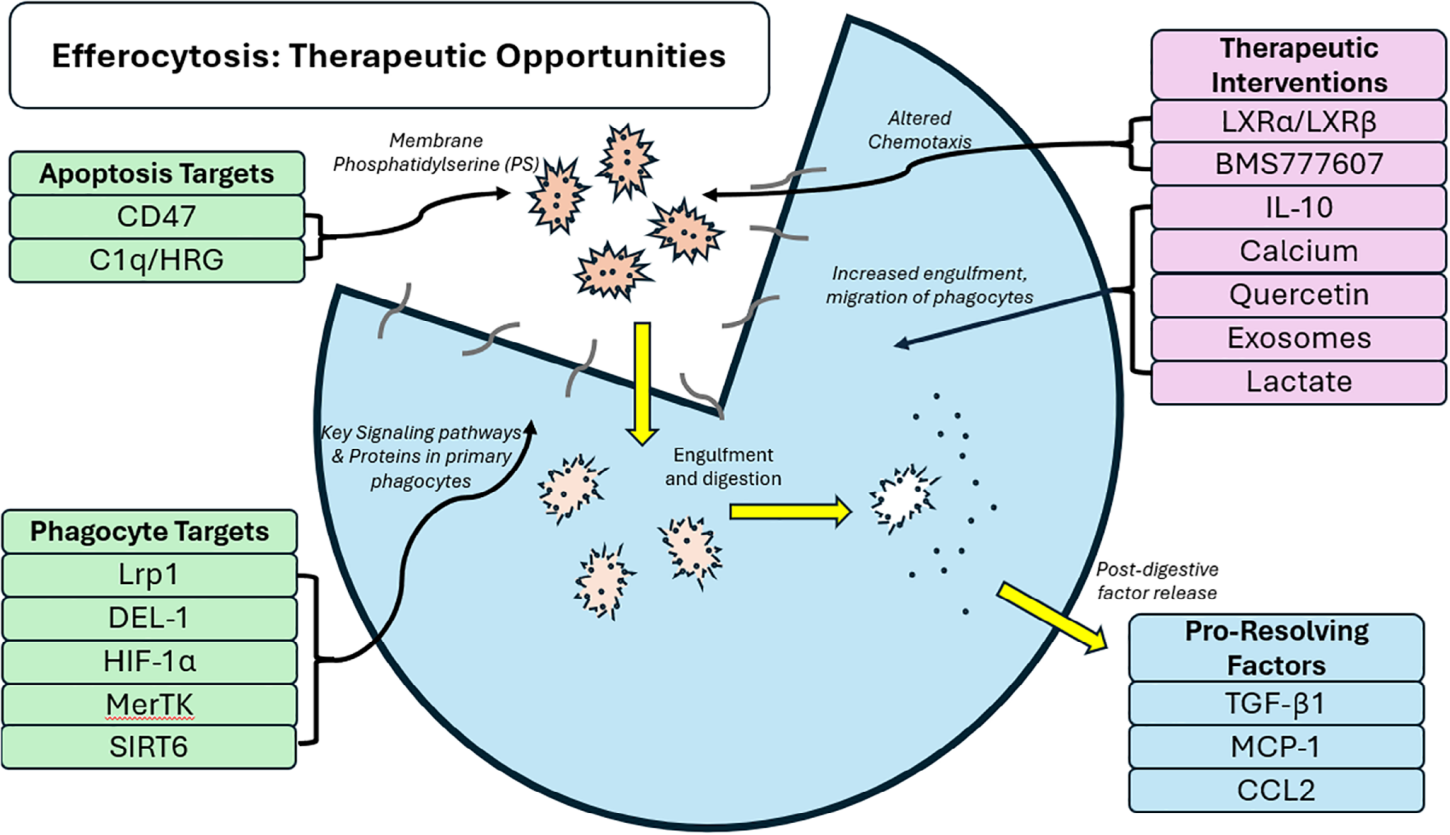
Therapeutic opportunities in the efferocytosis cascade, and strategies for intervention.

As efferocytotic cleanup involves a complex interaction between cleanup cells, signals, and apoptotic cell populations, any number of mechanisms can be disrupted, lead to impaired clearances. Therefore, interventions must be tailored to directly address the problem cogs in the efferocytotic machine – from empowering the macrophage “cleaners” to improve their appetite, to making the apoptotic cells more enticing targets for cleanup, several intervention targets are available (Figure 1).

### 4.1 Macrophage Targets

Macrophages are one of the primary drivers for efferocytosis thanks to their role as a professional phagocyte, and as such are prime therapeutic targets for interventions. It is generally understood that M2 macrophage polarization results in increased phagocytic activity as compared to the lower phagocytic capacity of M1 polarized macrophages [73, 74, 75]. However, the mechanism linking macrophage polarization to phagocytic activity is not well defined. Additionally, there is recent evidence that within the M1 and M2 macrophage populations, there exists phenotype heterogeneity [76, 77, 78] that further muddies the picture.

Receptor MerTK is recently gaining attention for its role in macrophage‐mediated efferocytosis. Deletion of MerTK has been shown to obliterate macrophage removal of apoptotic cells in various models [37, 79, 80], but therapies targeting the receptor complex still paint an unclear landscape. In the tumor environment, high expression levels of MerTK and efferocytosis have been shown to increase tumor progression, where anti‐MerTK therapeutics see more promise in anti‐tumor immunity [30, 31, 32]. This suggests the tumor's ability to harness efferocytosis to evade immune surveillance, metastasize, and drive angiogenesis. Rapid removal of apoptotic cell populations is shown to generate an increased immunosuppressive effect, advancing tumor growth in breast cancer [81]. As MerTK expression in various tissues is altered in different diseases and age states [82], this posits a significant number of circumstances in which its role governing efferocytosis can be investigated.

**FIGURE 2:**
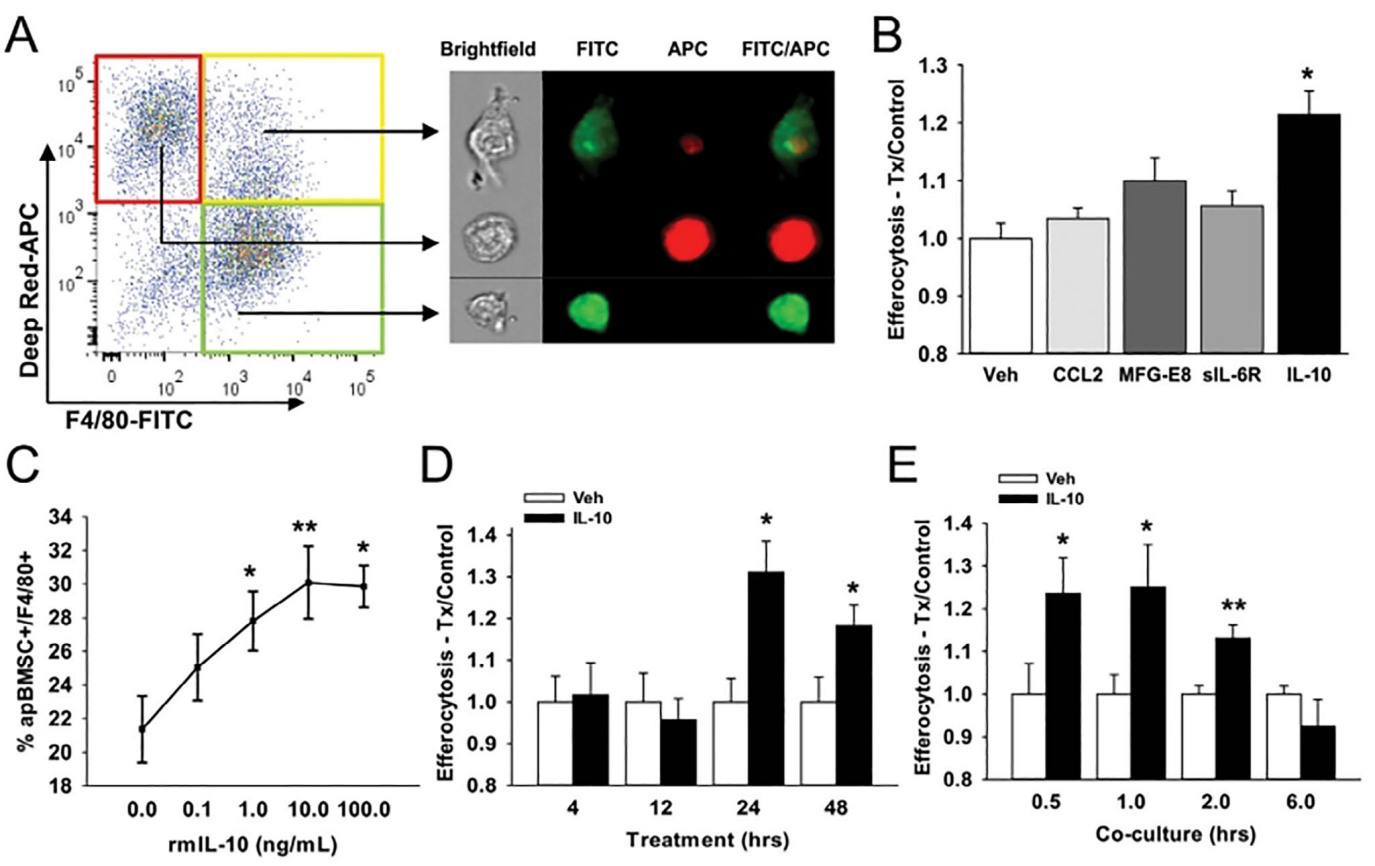
Efferocytosis of apoptotic bone marrow stromal cells (apBMSCs) as evaluated by flow cytometry. Macrophages with internalized apBMSCs (yellow gate, A). Adapted with permission [43]. Copyright 2016, John Wiley and Sons.

The signaling environment plays a key role in macrophage polarization and activity. Bone marrow‐derived macrophages treated with anti‐inflammatory cytokine IL‐10 showed increased efferocytotic removal of apoptotic bone marrow stromal cells (apBMSCs) [43]. After IL‐10 treatment, engulfment of apBMSCs was increased in a time and dose‐dependent manner, as evident by macrophages associated with apBMSCs quantified by flow cytometry (Figure 2). It was overall found that IL‐10 treatment increased M2 macrophage polarization and cleared apoptotic BMSCs in a STAT3 phosphorylation‐dependent manner.

For natural interventions, evidence suggests that macrophages can be “educated” by exosomes from BMSCs. Zhang et al. describe exosomal‐induced anti‐inflammatory polarization of macrophages that demonstrated significantly improved efferocytosis of apoptotic cellular debris in a mouse lupus nephritis model [83]. Interestingly, not only did macrophages show increased phagocytic engulfment, but in vivo noted an increase in recruitment of regulatory T‐cells, further bolstering local phagocytic activity.

Hypoxia‐driven cellular behavior is a well‐studied phenomenon affecting outcomes in bone regeneration, with several therapeutic opportunities available [84, 85, 86], but the impact of hypoxic conditions in efferocytosis is thus far unclear. Norris et al. describe hypoxia‐induced acceleration of apoptotic neutrophil efferocytosis in M2 macrophages via specialized pro‐resolving mediators (SPMs) [87]. Macrophage incubation in physiologic hypoxia conditions exhibited increased efferocytosis of apoptotic neutrophils. It was found that physiologic hypoxia induction enhanced SPM production and expression of hypoxia‐inducible factor 1‐a (HIF‐1α), which subsequently resulted in significantly increased M2 macrophage efferocytosis of senescent human red blood cells.

HIF‐1α shows additional evidence as an important player in its role in efferocytosis of apoptotic prostate cancer cells by BM macrophages, as shown by Mendoza‐Reinoso and coworkers [88]. Their findings demonstrate that BM macrophages stabilize and promote nuclear translocation of HIF‐1α, which induces expression of pro‐inflammatory macrophage migration inhibitory factor (MIF)—which is known to lead to the activation and secretion of pro‐tumorigenic cytokines [89, 90].

Furthermore, a hypoxia environment induces anaerobic glycolysis and accumulation of lactate, which plays an important role in efferocytosis‐induced macrophage proliferation (EIMP) and subsequent tissue repair. Lactate stimulates macrophage secretion of pro‐resolving factors and, in turn, promotes continued efferocytosis, where a single macrophage sequentially uptakes multiple apoptotic cells [91, 92]. Ngai et al. demonstrate that lactate increases the stabilization of Myc protein through SIRT‐1 mediated deacetylation, downstream of a PKA–AMPK–NAD^+^ pathway, leading to the promoted EIMP and expansion of pro‐solving macrophages [91]. Moreover, lactate stimulates efferocytosis‐induced macrophage glycolysis by upregulation of efferocytosis receptors, including MerTK and LRP1, and facilitates the continual efferocytosis process [93, 94]. Morioka et al. report that increased release of lactate by efferocytic phagocytes, where efferocytosis is modulated through expression of solute carrier (SLC) family of membrane transport proteins, subsequently influences non‐engulfing naive macrophages in the tissue microenvironment toward anti‐inflammatory polarization [95]. Additionally, efferocytosis‐induced lactate acts in a paracrine manner to mediate immunosuppressive effects of efferocytosis via upregulating expression of anti‐inflammatory genes, *Tgfb* and *Il10*, and M2‐like genes *Vegfa*, *Mgl1*and *Cd206* [96]. Moreover, lactate has been widely studied and shown to improve the regenerative and reparative strategies in various tissues and animal models, including spinal cord and brain injury, degenerative intervertebral disc, and bone tissue defects, etc [97, 98, 99]. Deng et al. discuss that lactate regulates the osteogenesis process through activating Olfr1440 and accelerates bone healing [100]. In addition to the direct promoting osteogenic differentiation, our unpublished data suggests that lactate also plays a significant role in modulation of macrophage function and osteoclastogenesis which are both known to be important for bone regeneration. Besides, lactate based and related polymers, including poly(lactic‐co‐glycolicacid) and poly‐L‐lactic acid, have been employed as templates to facilitate macrophage uptake and modulate the inflammatory microenvironment by reprograming inflammatory macrophages to the pre‐regenerative phenotype, providing strategies for atherosclerosis therapy and nerve regeneration [101, 102]. While lactate emerges as a promising target for tissue regeneration, its potential roles in efferocytosis to facilitate bone recovery process remain largely unknown. The underlying mechanism of lactate on macrophage‐mediated efferocytosis will be targeted in further studies to evaluate its effects on efferocytosis from a regenerative perspective.

### 4.2 Apoptotic Cellular Targets

While the strategies targeting phagocytes see some success in resolving inflammation by managing efferocytosis, a lesser used but emerging alternative strategy is to target the apoptotic cells. In most diseases, a dysregulated efferocytosis environment will see challenges to both the phagocyte and apoptotic cell populations concurrently. However, most studies involving efferocytosis are aimed at the phagocyte, and as such, less is known about the impact of the changing apoptotic cell populations in disease, trauma, and aging.

**FIGURE 3:**
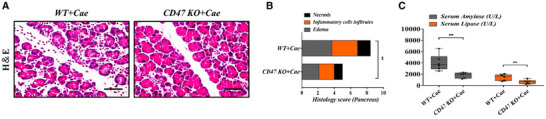
Conventional CD47 knockout improves efferocytosis in AP mice. Adapted with Permission [105]. Copyright 2024, Elsevier.

CD47 (cluster of differentiation 47) is a transmembrane protein that is well‐associated with apoptosis in humans. As a key marker signaling “don't eat me”, it is commonly overexpressed in various types of cancer [103, 104], where it helps enable cells to avoid programmed death. Consequently, resolution of inflammation is delayed with CD47 overexpression, and therefore is an attractive therapeutic target for the treatment of cancer and inflammatory diseases. Zhu et al. describe one such example in acute pancreatitis by defining the TRIM28‐miR133a‐CD47 signaling axis, where CD47 inhibition resulted in promoted efferocytosis of apoptotic acinar cells by macrophages in mice (Figure 3) [105].

Another study noted that increased CD47 levels of apoptotic osteoblasts led to reduced efferocytosis in aged mice, which could be effectively treated to restore phagocytic take up by increased SIRT6 expression via delivery of miRNA‐loaded apoptotic vesicles [34].

Li and coworkers describe the therapeutic effect of plant‐derived flavonoid Quercetin on antitumor immunity via its role in decreasing the expression of CD47 [106]. It was found that quercetin increased M1 macrophage polarization and recruitment of CD5+T and CD8+T cells, owing to improved phagocytosis due to inhibited CD47 expression in mouse and human melanoma cells. While M2 macrophages are typically thought of as the primary drivers of efferocytosis, the authors noted increased M1 macrophage proliferation in this study. Additionally, no evidence was able to suggest which of the cells, macrophages or T cells, played the primary role of phagocyte in their model.

Other surface markers can play a role in evasion of immune surveillance. For instance, engulfment receptors on phagocytes can be bridged with membrane proteins like complement component 1q (C1q) [107] and histidine‐rich‐glycoprotein (HRG) [108] in apoptotic cells. Changes in these proteins have been described in aged mice [109] and humans [110], but no follow‐up studies have been performed to evaluate their effects on efferocytosis or other phagocytic functions.

**FIGURE 4:**
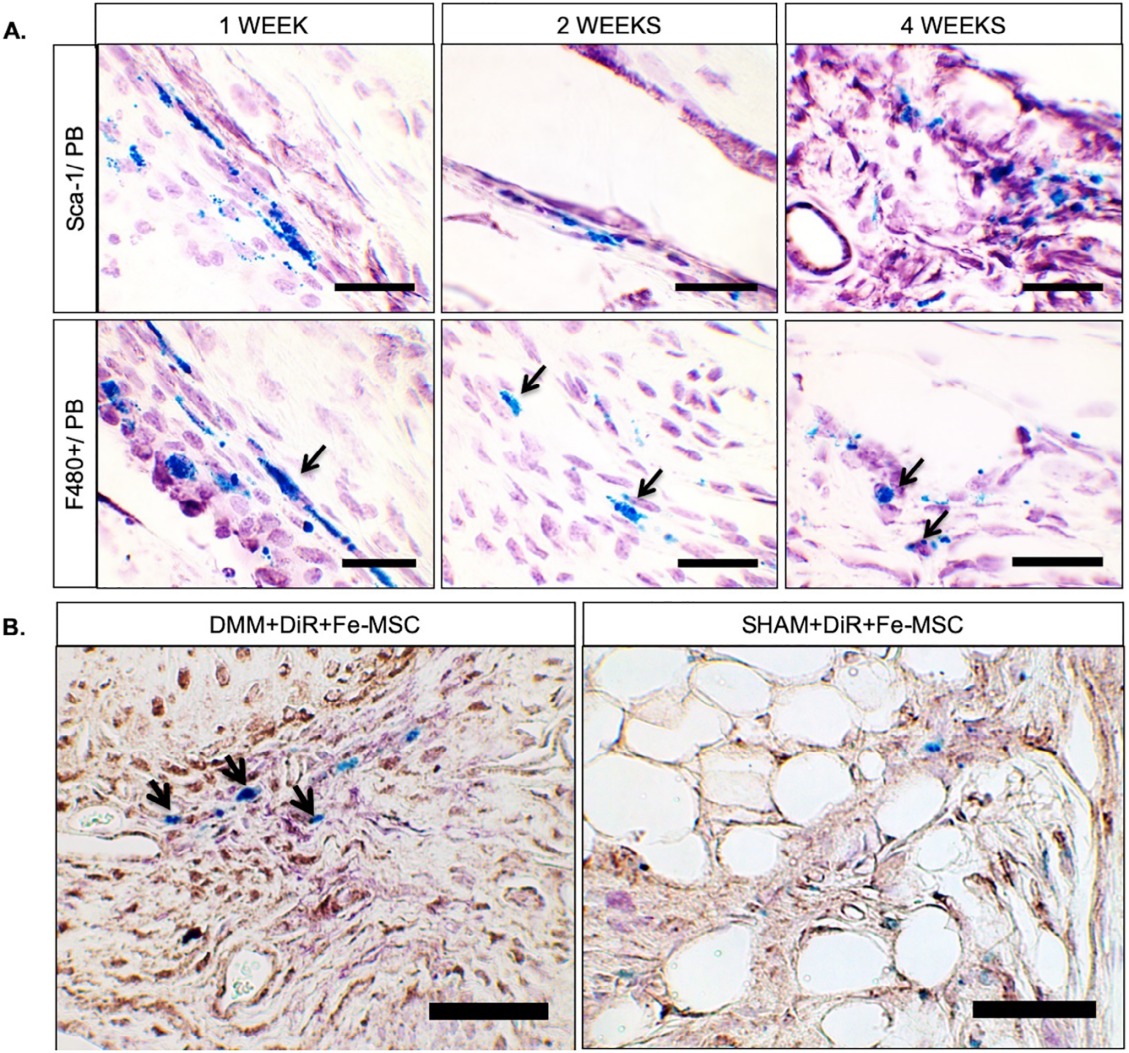
Co‐staining of F4/80 (purple) and Prussian blue (blue) suggests evidence of efferocytosis. Scalebar = 50 µm. Adapted with permission [112]. Copyright 2019, Public Library of Science (PLOS).

In osteoarthritis (OA) joints, macrophage count is elevated compared to healthy levels [111]. Reduced apoptotic debris can result in a loss of pro‐resolving factors necessary for inflammation resolution, driving the progression of OA, which is especially challenging in low cellular density tissues like cartilage. Hamilton and coworkers describe evidence for resolution of OA by delivery of transplanted MSCs labeled by superparamagnetic iron oxide nanoparticles (Ferumoxytol) [112]. MSC delivery improved M2 macrophage polarization and residual Prussian blue staining in macrophages (Figure 4) suggested phagocytic uptake of MSCs post‐injection, indicating efferocytosis could help resolve and improve OA conditions in mice.

Similarly, in Leukocyte adhesion deficiency Type 1 (LAD1) periodontitis, a reduced neutrophil population can cause recurrent bacterial infection and chronic inflammation due to the loss of pro‐resolving factors from low efferocytosis. Kajikawa et al. demonstrated that bone loss in a mouse LAD1 periodontic model can be restored via pharmacologic intervention with synthetic agonists LXRα/LXRβ and PPARβ/δ, mimicking the anti‐inflammatory function of efferocytosis [113]. After treatment, alveolar bone height loss was restored, and pro‐inflammatory markers IL‐23 and IL‐17 were decreased, demonstrating the effectiveness of pharmacologic approaches at mimicking apoptosis. As the role of apoptotic populations becomes clearer over time, it will open the door for more therapeutic targets as different disease and illness states become elucidated.

## 5 Engineering Efferocytosis with Biomaterials

Biomaterials‐based approaches to bone defect repair aim to improve tissue regeneration with minimal use of exogenous bioactive factors and stem cells. With a high degree of tunability, biomaterials in today's age can exert significant influence over stem cell and macrophage behavior in vivo, leading to increasingly favorable results in the field of regenerative medicine for bone repair. Numerous well‐documented techniques to develop material scaffolds exist, from hydrogels [114, 115] and self‐assembled materials [116, 117] to biomimetic strategies including phase separation and electrospinning [118, 119], and recently, the rapidly evolving 3D printing technologies. These techniques usually have well‐defined, rigorously tested models centered around the osteogenic differentiation of stem cells, aimed at rapid translation to in vivo research. However, these approaches are typically agnostic toward efferocytosis due to their scope, lack of effective in vitro models, and a recent growing understanding of its role in injury and disease resolution.

More recently, immunomodulatory materials are one example of biomaterials that seek to leverage innate immunity in their favor for better outcomes in tissue repair [120, 121]. For example, it is well‐studied that manipulating the shape of macrophages via controlling the biomaterial microstructure can influence their polarization and subsequent downstream activity [122, 123, 124]. Macrophage polarization can also be influenced by other means, such as growth‐factor eluting materials irrespective of morphology [125, 126]. However, biomaterials‐based interventions infrequently observe downstream effects on phagocytic activity or apoptotic cell populations and the resulting impact on efferocytosis. Efferocytosis promoting materials are a recently emerging strategy that seeks to enhance or recover dysregulated repair environments in the bone niche—either by influencing phagocyte activity or altering the perception and activity of apoptotic cells (Table 2).

### 5.1 Biomaterials Targeting Macrophages

Loss of homeostatic environment is one driving factor to reduced efferocytosis in bone injury. A secondary contributor in reduced efferocytic activity is oxidative stress in phagocytes. Wang et al. describe a biomimetic polydopamine coated gelatin/polylactic acid (PLA) sponge that contained apoptosis‐mimetic CeO_2_ (AM/CeO_2)_ nanoenzymes to mimic a pro‐efferocytosis microenvironment and provide antioxidant protective effects [127]. AM/CeO_2_ coated scaffolds demonstrated effective repair of bone defects and activated continual efferocytosis of macrophages. This ultimately led to improved ROS scavenging and phagocytic take up of nanoenzymes compared to controls, leading to accelerated bone repair.

Specialized pro‐resolving lipid mediator (SPM) LipoxinA4 (LXA4) plays a key role in inflammatory resolution during endometriosis [128], and the small molecule LXA4 agonist BML‐111 has shown similar inflammatory protective function in mice [129, 130]. However, its mechanism is unknown. It is also noted in literature that intracellular calcium levels play a significant role in efferocytosis [131, 132, 133], where certain physiologic conditions can impede phagocytes' ability to continually engulf cells. Sun et al. investigate the mechanism of LXA4 via a nanoparticle approach ‐ synthesizing BML‐111 loaded CaCO_3_ nanoparticles that could effectively release dual‐release calcium and BML‐111 in an acidic environment [134]. The resulting nanoparticles were able to strengthen the phagocytic activity of RAW264.7 macrophages and slow the progression of ectopic lesions in a surgically induced endometriosis mouse model, thanks to decreased inflammatory factors like IL‐1β in the abdominal cavity.

Biomaterial approaches are also shown to be effective at selectively inhibiting efferocytosis – a therapeutic strategy with promise in cancer treatment. Wu and colleagues demonstrate release of small molecule (BMS777607) from nanoprecipitation‐encapsulated PLGA (nano‐BMS), which resulted in phagocytic suppression, and subsequent direction of apoptotic cells to secondary necrosis [135]. BMS777607 selectively interfered with phosphatidylserine‐dependent efferocytosis, allowing apoptotic cells to be exposed to immune surveillance. When combined with the common chemotherapy drug Cisplatin, nano‐BMS encouraged M1 polarization of BMDMs concurrently incubated with apoptotic CT26 cells, which eventually resulted in tumor growth suppression in mice (Table 2).

**TABLE 2:** Overview of biomaterials‐based strategies to engineer efferocytosis, based on their target in the efferocytosis pathway and the intended mechanism.

Biomaterial type	Efferocytosis target	Target mechanism
Polydopamine‐coated gelatin/PLA sponge [127]	Macrophages	Oxidative stress pathway
CaCO_3_ nanoparticles [134]	Macrophages	LipoxinA4 (LXA4) inflammatory resolution
PLGA nanospheres [135]	Macrophages	Phosphatidylserine receptors
Hyaluronic acid hydrogel microspheres [136]	Apoptotic/Senescent cells	Phosphatidylserine receptors in senescent chondrocytes
Hyaluronic acid methacrylate hydrogel microspheres [137]	Macrophages/Apoptotic cells	CCL19‐specific receptor CCR7 chemotaxis
Silica Dioxide‐loaded pH‐reponsive hydrogel [138]	Apoptotic cells	Neutrophil infiltration
Gelatin Methacryloyl (GelMA) hydrogel [139]	Apoptotic neutrophils	Neutrophil membrane stability

### 5.2 Biomaterials Targeting Apoptotic Cells

**FIGURE 5:**
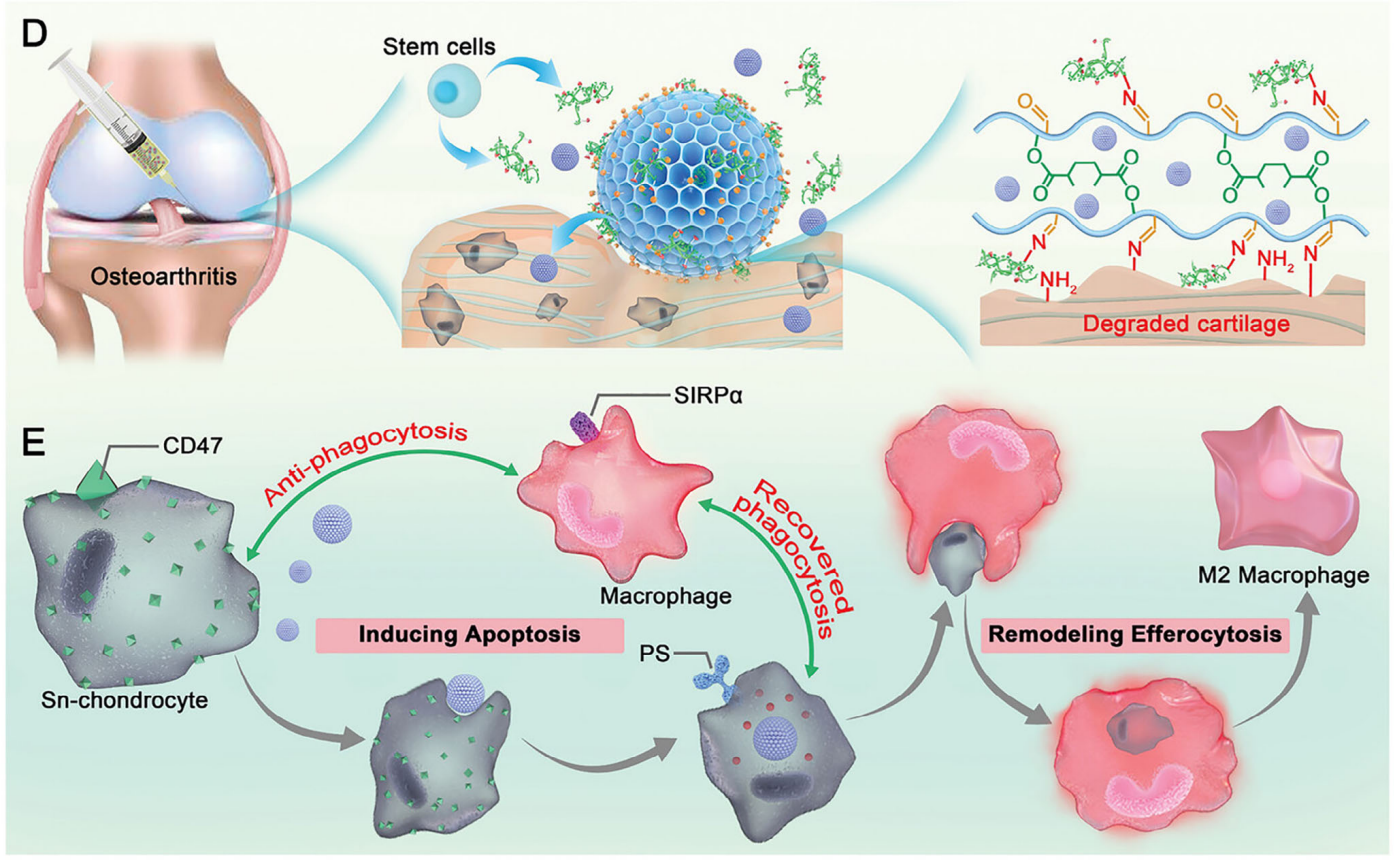
Proposed delivery technique and mechanism of efferocytosis improvement from A‐Lipo/PAHM microspheres. Adapted with permission [136]. Copyright 2024, John Wiley and Sons.

Insufficient levels of or irregularities in apoptosis lead to a lack of activity from phagocytes, which can be rendered by inducing sufficient apoptosis of local cells. As mentioned previously, OA joints are prime cases for environments like these. Senescent cell populations are especially prone to irregular apoptosis patterns, making them effective targets for biomaterials‐mediated therapeutics. Xiong and coworkers demonstrated reversal of cartilage senescence and accelerated cartilage repair by use of modified hyaluronic acid hydrogel microspheres loaded with pro‐apoptotic liposomes (A‐Lipo) [136]. A‐Lipo was combined with platelet‐derived growth factor (PDGF‐BB) to recruit stem cells and hunt senescent chondrocytes, and induce apoptosis (Figure 5). This promoted reversal of cartilage senescence in OA when localized to cartilage lesions, showing great promise for as a potential therapeutic option.

Another study utilized hydrogel microspheres in an OA model to selectively recruit and induce apoptosis of M1 macrophages, effectively remodeling efferocytosis for macrophage population balancing [137]. Wang et al. describe complex clondronate liposomes modified with CD16/32 antibodies, loaded into CCL19 surface‐modified microspheres that recruit M0 and M1 macrophages and induce apoptosis. This cleverly reduced pro‐inflammatory macrophage numbers while simultaneously providing the “find me” signals that are ordinarily lacking in an OA model, balancing the macrophage count and accelerating treatment.

**FIGURE 6:**
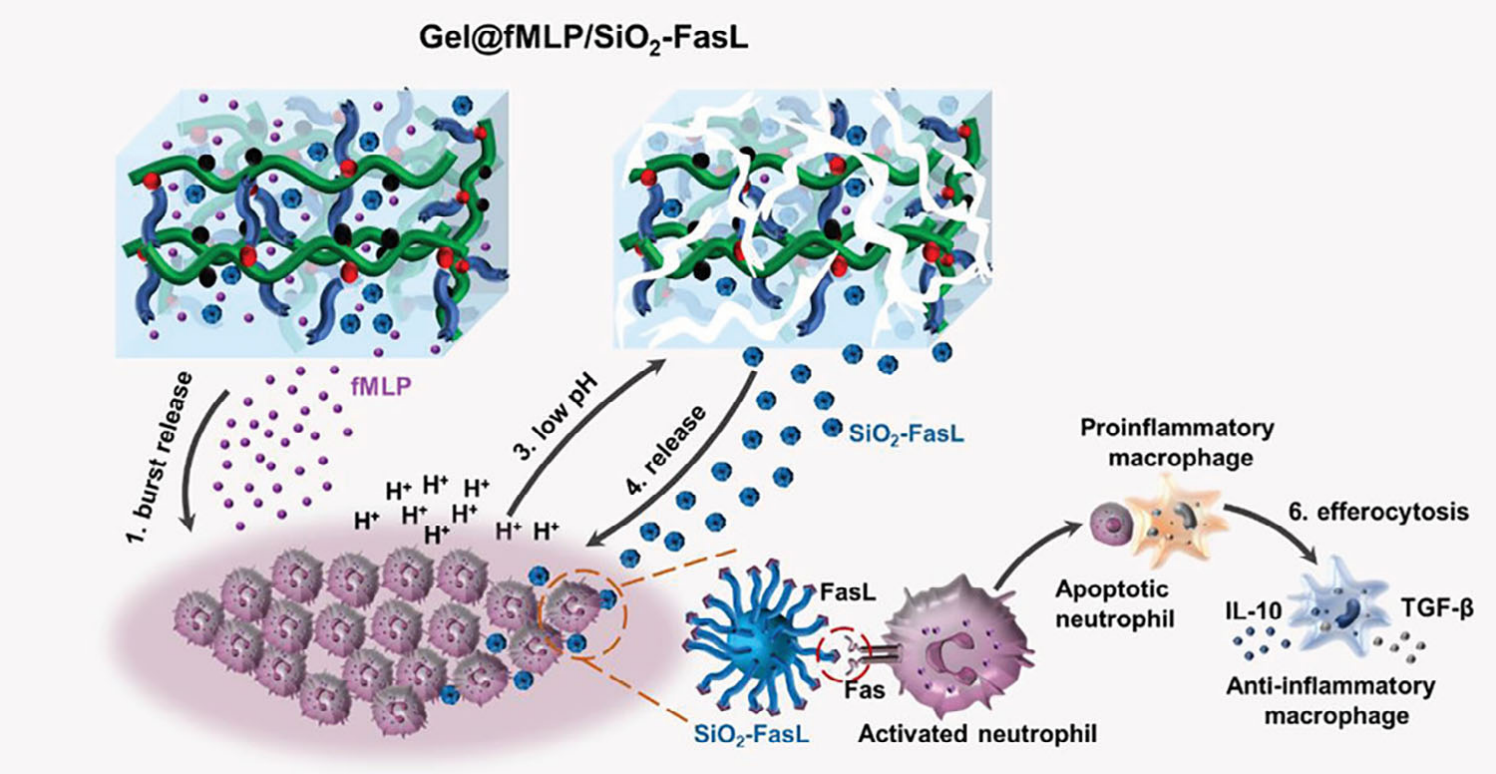
Schematic Illustration of Silica Dioxide nanoparticles release for transient efferocytosis activation. Adapted with permission [138]. Copyright 2022, John Wiley and Sons.

Neutrophils are transiently expressed in high volume during the inflammatory response, releasing a variety of growth and pro‐angiogenic factors, and are critical components in kickstarting the regeneration process. However, they must be effectively cleared via efferocytosis for the continuation of healing. Liu et al. utilize a pH‐responsive hydrogel (Figure 6) to reprogram the inflammatory process and control neutrophil infiltration and clearance to improve critical‐size calvarial bone defect repair [138]. Macrophage M2 markers improved, and neutrophil apoptotic populations decreased with the release of FasL‐conjugated silica dioxide nanoparticles – evidence of efferocytosis; however, more direct evidence of engulfment was not studied. Zhuo and coworkers describe a multifunctional hydrogel to promote more effective neutrophil apoptosis in a periodontitis model [139]. Exogenously providing apoptotic neutrophil byproducts can help elicit efferocytosis, as the authors describe an antimicrobial peptide MP196 combined with gelatin methacryloyl (GelMA) and neutrophil exosomes. Improved M2 polarization of macrophages in periodontal tissue was noted, alongside an increase in phagocytic capacity. In a periodontal rat model, their novel material also led to diminished alveolar bone resorption, indicating it has strong potential as a potential therapeutic strategy to modulate efferocytosis.

## 6 Challenges and Future Perspectives

While efferocytosis is a recently coined term, the umbrella topic of inflammatory resolution has been studied in various chronic diseases and illnesses for decades. Only more lately has significant attention been paid to the role of phagocytic removal of apoptotic debris in tissue regeneration, as it is becoming evident of the large role efferocytosis plays. However, within the context of current research in bone, some clear challenges remain. Both phagocyte and apoptotic cell populations are highly transient, leading to challenges in in vivo observation. Additionally, the rapid cell turnover in the bone environment creates difficulty in establishing reliable in vitro models. The evident ability of both professional and non‐professional phagocytes to perform efferocytosis must be more clearly studied, as it is becoming more clear that a significant number of different cell types possess phagocytic machinery and may contribute in efferocytosis in bone.

To date, no standardized method of quantifying efferocytosis is commonly used. Recent studies primarily utilize indirect evidence, such as fluorescence and other evidence of cellular debris found inside of suspected phagocytes. However, some attempts to establish assays have been made via counting methods [140] or flow cytometry [141], but their effectiveness and adaptability to different types of phagocytes remains to be studied. Additionally, the differences in efferocytosis activity between professional and non‐professional phagocytes should be taken into consideration.

While its essential role in immune function is becoming increasingly clear, efferocytosis is susceptible to hijacking by oncogenic forces like many other biological systems. Continual apoptotic cell clearance via efferocytosis is a usual mechanism in tumorigenic environments, blocking antitumor immunity. There is some evidence that anti‐efferocytosis therapeutic strategies lead to antitumor outcomes via induction of secondary necrosis, but little evidence on safety and long‐term side effects of these interventions currently exists.

Finally, while the individual aspects of efferocytosis (e.g. the phagocyte, apoptotic cells) have seen study, more holistic approaches should be considered. Rarely is a single element at fault in a dysregulate inflammatory environment, with its many moving parts playing small to large roles in the non‐resolution of inflammation. Many of the current strategies in literature are aimed at therapeutically intervening at singular actors in the inflammatory cascade, and as such may produce drastically inferior improvements to resolving inflammation via efferocytosis.

As the world's population age increases and life expectancy continues to rise, efferocytosis‐aware therapeutics could be a potentially powerful tool to combat aging‐related and aging‐complicated disease in the clinic moving forward. There is clear evidence that immunomodulatory biomaterials are strong tools for improved bone healing outcomes, and incorporating efferocytosis‐specific concerns is a logical next step.

Overall, efferocytosis is a rapidly evolving topic that is seeing many links to chronic inflammation, disease, aging, and is certainly a critical avenue of study for the future development of therapeutics to improve public health.

## Conflicts of Interest

The authors declare no conflicts of interest.
